# Novel CXCL13 transgenic mouse: inflammation drives pathogenic effect of CXCL13 in experimental myasthenia gravis

**DOI:** 10.18632/oncotarget.6885

**Published:** 2016-01-11

**Authors:** Julia Miriam Weiss, Marieke Robinet, Revital Aricha, Perrine Cufi, Bérengère Villeret, Frida Lantner, Idit Shachar, Sara Fuchs, Miriam C. Souroujon, Sonia Berrih-Aknin, Rozen Le Panse

**Affiliations:** ^1^ INSERM U974, Paris, France; ^2^ CNRS FRE3617, Paris, France; ^3^ Sorbonne Universités, UPMC University Paris 06, Paris, France; ^4^ AIM, Institut de Myologie, Paris, France; ^5^ Department of Immunology, Weizmann Institute of Science, Rehovot, Israel; ^6^ Open University of Israel, Raanana, Israel

**Keywords:** chemokine, autoimmunity, thymus, B cells, CXCL13-CXCR5, Immunology and Microbiology Section, Immune response, Immunity

## Abstract

Abnormal overexpression of CXCL13 is observed in many inflamed tissues and in particular in autoimmune diseases. Myasthenia gravis (MG) is a neuromuscular disease mainly mediated by anti-acetylcholine receptor autoantibodies. Thymic hyperplasia characterized by ectopic germinal centers (GCs) is a common feature in MG and is correlated with high levels of anti-AChR antibodies. We previously showed that the B-cell chemoattractant, CXCL13 is overexpressed by thymic epithelial cells in MG patients. We hypothesized that abnormal CXCL13 expression by the thymic epithelium triggered B-cell recruitment in MG. We therefore created a novel transgenic (Tg) mouse with a keratin 5 driven CXCL13 expression.

The thymus of Tg mice overexpressed CXCL13 but did not trigger B-cell recruitment. However, in inflammatory conditions, induced by Poly(I:C), B cells strongly migrated to the thymus. Tg mice were also more susceptible to experimental autoimmune MG (EAMG) with stronger clinical signs, higher titers of anti-AChR antibodies, increased thymic B cells, and the development of germinal center-like structures. Consequently, this mouse model finally mimics the thymic pathology observed in human MG.

Our data also demonstrated that inflammation is mandatory to reveal CXCL13 ability to recruit B cells and to induce tertiary lymphoid organ development.

## INTRODUCTION

CXCL13 is a chemokine mainly expressed by secondary lymphoid tissues, such as lymph nodes, spleen and gut-associated lymphoid tissues. In contrast to many other chemokines, CXCL13 possesses a unique receptor named CXCR5 [1, 2] and is known to fulfill two distinct functions. One is its contribution to the formation of secondary lymphoid organs, which was demonstrated in transgenic mice lacking CXCL13 or CXCR5 [3, 4]. These mice are characterized by malformed Peyer's patches, a disrupted spleen structure and a lack of most lymph nodes. Secondly, CXCL13 is especially known for its strong homing effect on B cells [2] and on a small subset of CD4 T cells, follicular helper T (TFH) cells [1], to secondary lymphoid organs but also inflamed tissues [3, 5, 6]. Once CXCL13 has recruited lymphoid cells to target tissues, it guides them to get organized in B-cell follicles and in case of an immune response, B cells proliferate to form germinal centers (GCs) [7]. The positioning of B and TFH cells in GCs strongly depends on CXCL13-CXCR5 interactions [8].

Inflammatory conditions, including autoimmunity, persistent infections and cancer are often associated with the accumulation of lymphoid cells leading to ectopic GC development transforming the inflamed tissue into a tertiary lymphoid organs [6]. The abnormal overexpression of CXCL13 is usually described in these inflammatory tissues. For example, CXCL13 is detected in salivary glands of Sjogren's syndrome patients and is associated with local GC formation [9]. In multiple sclerosis, CXCL13 is increased in the cerebrospinal fluid and its level correlates with cerebral B-cell infiltrations, antibody production and relapse rates [10]. Acquired Myasthenia Gravis (MG) is a neurological disease mainly caused by autoantibodies against the acetylcholine receptor (AChR) [11] leading to muscle disabling fatigability. MG is a prototype autoimmune disease and if the target organ is the muscle, the effector organ is the thymus [12]. The MG thymus includes all the components of the anti-AChR response: AChR expression by thymic epithelial cells (TECs) and myoid cells [13], presence of B cells producing anti-AChR antibodies [14] and of anti-AChR autoreactive T cells [15]. In early onset MG, the thymus displays all the characteristics of a tertiary lymphoid organ [6] with an increased amount of B cells and ectopic germinal centers (GCs), whose number correlates positively with anti-AChR titers [16]. In the thymus of MG patients, CXCL13 is overexpressed by medullary TECs [17, 18, 19] and several indications suggest that CXCL13 could be involved in the infiltration of B cells: 1) thymic extracts from MG patients have a strong chemoattractive effect on B cells, 2) this effect is reduced when using anti-CXCL13 blocking antibodies and 3) the thymus of MG patients under corticotherapy shows a normalized level of CXCL13 together with a reduced number of GCs [17]. Moreover, thymic and serum levels of CXCL13 were shown to correlate with disease severity [17, 19, 20].

We therefore hypothesized that increased production of CXCL13 by TECs could be sufficient to induce B-cell recruitment. To this end, we created a novel transgenic (Tg) mouse with a keratin 5 (K5) driven expression of CXCL13. Our results show that thymic overexpression of CXCL13 under steady state condition did not induce B-cell recruitment to the thymus. However, under inflammatory conditions, CXCL13 overexpression was able to trigger B-cell migration towards the thymus. Immunization with purified AChR showed that transgenic mice were more susceptible to experimental autoimmune MG (EAMG) with increased clinical signs, elevated levels of anti-AChR antibodies and in some cases the development of thymic GC-like structures. Altogether this EAMG model recapitulated better the human pathology than the classical model which does not show thymic pathology. Our data suggest that thymic follicular hyperplasia is the result of combined features, including overexpression of CXCL13 and increased inflammation.

## RESULTS

### Thymic overexpression of CXCL13 in K5-CXCL13 transgenic (Tg) mice

We created Tg mice that carry the gene for murine CXCL13 under the control of the K5 promoter (Supplemental Figure S1), in order to mimic the overexpression of CXCL13 in medullary epithelial cells of MG thymus. These mice did not exhibit a particular phenotype and appeared similar to wildtype (WT) mice as they grew up and bred as WT mice. They did not show evident motor impairment compared to WT mice but had less muscle strength. With the grip strength apparatus, 6-week-old WT (*n* = 22) and Tg (*n* = 22) female mice developed a strength of 138.6gr and 121.5gr (*p* < 0.001), respectively.

**Figure 1:**
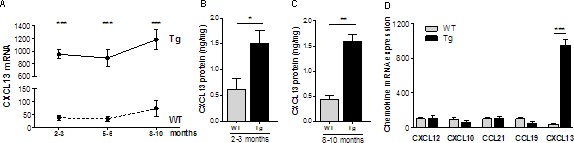
Chemokine expression in the thymus of K5-CXCL13 Tg mice **A.** CXCL13 mRNA level in the thymus of WT and K5-CXCL13 Tg mice at different age (*n* = 7-21 per group). PCR results were normalized to GAPDH. **B.**-**C.** CXCL13 protein levels measured by ELISA in WT and Tg mice at different age (*n* = 5-6 per group). **D.** Chemokine mRNA expression in the thymus of 2- to 3-month-old WT and Tg mice. CXCL12, CXCL10, CCL21, CCL19 expression (*n* = 5 and 6 for WT and Tg, respectively) were compared to CXCL13 (*n* = 17 and 21 for WT and Tg, respectively). PCR results were normalized to GAPDH. p-values were assessed by the Mann-Whitney test and only *p*-values < 0.05 are indicated (*p < 0.05; **p < 0.01; ***p < 0.001).

We first analyzed CXCL13 mRNA expression in the thymus of Tg mice aged from 2 to 10 months. While CXCL13 mRNA levels slightly increased with age in both WT and Tg mice, the levels in Tg mice were significantly higher compared to WT mice (Figure 1A). At the protein level, we also clearly observed higher expression of CXCL13 in the thymus of Tg mice with a 2.5 to 3.7 times increase in young and old mice, respectively (Figure 1B-1C).

To examine whether the expression of other chemokines was altered in the thymus of K5-CXCL13 Tg mice, we investigated chemokines that are known to be expressed in the thymus and that are dysregulated in the hyperplastic thymus of MG patients: CXCL12 [21], CXCL10 [22], CCL21 and CCL19 [18] [23]. By RT-PCR, we did not observe changes between WT and Tg mice for the expression of these chemokines (Figure 1D).

We also analyzed the mRNA expression level of CXCL13 in other organs beside the thymus. We selected organs known to express K5, such as the skin and salivary glands. As expected, we observed a strongly increased expression of CXCL13 in the thymus but also in salivary glands and an even higher expression in the skin (Supplemental Figure S2). As we were interested in using this Tg mouse model for studies related to MG, we focused our attention on the thymus. Altogether our results clearly confirmed the specific overexpression of CXCL13 in the thymus of Tg mice at the mRNA and protein levels.

### Thymic structure and cell populations in K5-CXCL13 Tg mice

**Figure 2:**
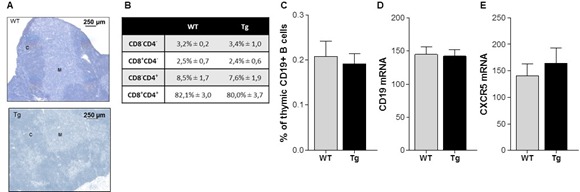
Thymic structure and proportion of T and B cells in K5-CXCL13 Tg mice A. Representative hematoxylin staining of 7 μm-thick thymic sections of 6 week-old Tg and WT mice with apparent cortical (C) and medullary (M) regions. **B.** Flow cytometry analyses of the percentage of thymocyte subpopulations in WT and Tg mice: double negative (CD4-CD8-), double positive (CD4+CD8+), CD4 or CD8 single positive (CD4+CD8− or CD4−CD8+) thymocytes (WT, *n* = 6 and Tg, *n* = 11). C. Flow cytometry analysis of the percentage of B cells (CD19+ cells) in the thymus of WT (*n* = 6) and Tg (*n* = 10) mice. (D-E) RT-PCR analysis of CD19 and CXCR5 mRNA expression in the thymus of WT (*n* = 11) and Tg (*n* = 5) mice. PCR results were normalized to GAPDH. p-values were assessed by the Mann-Whitney test but no significant differences were measured.

Compared to age-matched WT mice, K5-CXCL13 Tg mice did not show any differences in thymus weight (data not shown). A staining of thymus sections with hematoxylin showed that medullary and cortical regions were well preserved indicating that the thymic structure was not altered in Tg mice (Figure 2A). Analyzing the proportion of CD4 and CD8 thymic subpopulations by flow cytometry, we did not observe differences between Tg and WT mice suggesting that thymopoïesis was not affected in Tg mice (Figure 2B).

CXCL13 acts especially as a B-cell chemoattractant, as B cells express high levels of its receptor CXCR5 [2]. We therefore analyzed the proportion of B cells in the thymus by flow cytometry using the pan-B cell marker CD19 and by RT-PCR analyzing CD19 mRNA expression. Surprisingly, we did not observe any obvious B-cell recruitment in the thymus of Tg mice compared to WT mice despite the high level of thymic CXCL13 in Tg mice (Figure 2C-2D), nor did we observe an increased expression of CXCR5 mRNA (Figure 2E).

Altogether, these results revealed that, despite high levels of CXCL13 in K5-CXCL13 Tg mice, the proportions of lymphoid cells in the thymus were not different from WT mice and unexpectedly no B-cell colonization of the thymus was observed.

### Increased levels of CXCL13 and decreased number of CXCR5+ cells in the blood of K5-CXCL13 Tg mice

**Figure 3:**
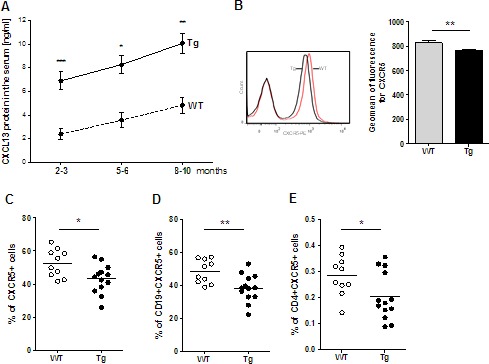
Alterations in CXCL13 serum level and circulating CXCR5^+^ cells in Tg mice **A.** ELISA quantification of CXCL13 concentrations in the serum of WT (*n* = 6-12 per group) and Tg mice (*n* = 9-12 per group) of different age. **B.**-**E.** Blood cells were labeled and analyzed by flow cytometry for CXCR5, CD4 and CD19. The percentage of cells and the geomean of fluorescence intensity were analyzed in the lymphocyte gate (determined according to the FSC/SSC characteristic profile of lymphocytes). These analyses were made on 2- to 3-month-old mice (WT, *n* = 10, Tg, *n* = 13). **B.** On the left, representative labeling of CXCR5^+^ cells for WT and Tg mice. On the right, geomean of fluorescence for CXCR5 on lymphoid cells for all mice. **C**.-**E**. Percentages of CXCR5^+^ lymphoid cells, CD19^+^CXCR5^+^ B cells and CD4^+^CXCR5^+^ T are shown. *p*-values were assessed by the Mann-Whitney test and only *p*-values < 0.05 are indicated (*p < 0.05; **p < 0.01; ***p < 0.001).

We also investigated if changes could be detected in the blood circulation of the Tg mice. The percentage of peripheral CD4 and CD8 T cells did not vary between Tg and WT mice but the number of B cells was significantly lower in Tg mice (Supplemental Figure S3A-S3C). Analyzing the level of CXCL13 in the serum of Tg mice, we observed an increase with age in both Tg and WT mice. In addition, CXCL13 levels in the serum of Tg mice were 2-3 times higher than in WT mice (Figure 3A). We then wondered if this high level of CXCL13 could bind to CXCR5 on circulating cells and alter their recruitment to peripheral organs. To investigate the consequences of the high CXCL13 serum level in Tg mice, we labelled peripheral blood cells for CXCR5. We observed a slight but significant decrease in the geomean of fluorescence for CXCR5 on lymphoid cells (Figure 3B) suggesting that the high level of serum CXCL13 could interact and lead to the internalization of CXCR5 on lymphoid cells. We also demonstrated a significant decrease in the percentage of CXCR5^+^ lymphoid cells in Tg mice (Figure 3C). Almost all B cells (around 95%) are CXCR5 positive and only a small percentage (less than 3%) of CD4 T cells are CXCR5^+^, which correspond to TFH cells. We observed a significant decrease of both CD19^+^CXCR5^+^ B cells and CD4+CXCR5+ T cells in the blood of Tg mice (Figure 3D-3E). These decreases suggest that a small percentage of CXCR5 positive cells could effectively be recruited to peripheral organs.

### Increased recruitment of B cells to the thymus of Tg mice in inflammatory condition

We then asked whether inflammatory conditions could induce changes in the thymus of Tg mice. We therefore challenged Tg mice with injections of Poly(I:C) which mimics dsRNA from viral infections and triggers a rapid inflammatory response in the thymus [24, 25].

**Figure 4:**
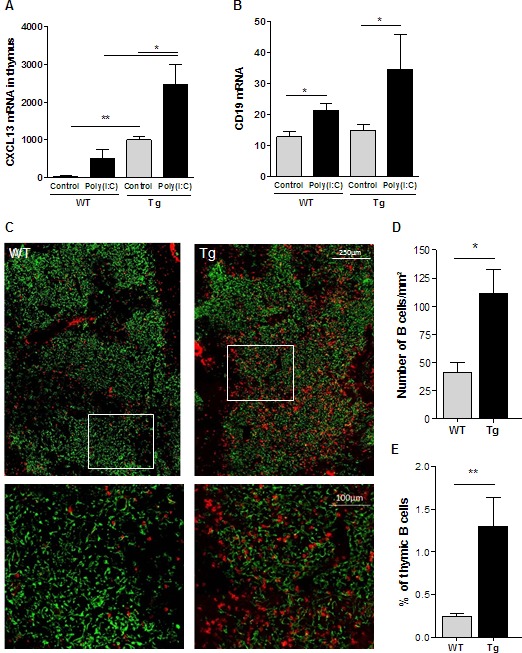
B-cell recruitment to the thymus of K5-CXCL13 Tg mice upon Poly(I:C) injections Poly(I:C) was i.p. injected to mice three times every two days and the thymus was analyzed 24 hours after the last injection. **A**.-**B**. RT-PCR analyses of CXCL13 and CD19 mRNA expression in the thymus of WT and Tg mice (*n* = 5-6) injected with physiological water (controls) or Poly(I:C). PCR results were normalized to GAPDH. **C.** Thymic sections from WT and Tg mice (*n* = 5-6) were stained with an anti-K5-FITC antibody (green) and a biotinylated anti-B220 antibody plus a streptavidin Alexa-Fluor-594 (red). Lower images correspond to magnification of areas delineated in white in upper images. **D.** The number of B cells was counted on the entire thymic section and normalized by the size of the section, which was assessed with the AxioVision software. **E.** Flow cytometry analysis of the percentage of thymic B cells (CD19^+^ cells) in WT and Tg mice (*n* = 5-6). *p*-values were assessed by the Mann-Whitney test and only *p*-values < 0.05 are indicated (*p < 0.05; **p < 0.01; ***p < 0.001).

In WT mice, Poly(I:C) injections are known to increase the thymic expression of diverse chemokines including CXCL13 [24, 25]. Here, we confirmed this effect of Poly(I:C) but the level did not reach the basal level of CXCL13 in control Tg mice (Figure 4A). However, we clearly observed that Poly(I:C) injections to Tg mice strongly induced the level of CXCL13 mRNA expression (Figure 4A). In parallel, we analyzed the level of CD19 mRNA, which reflects the proportion of B cells, and we observed a higher level of CD19 expression in Tg mice injected with Poly(I:C) compared to untreated Tg mice and compared to treated or untreated WT mice (Figure 4B). By immunohistochemistry, we clearly demonstrated that Poly(I:C) injections induced a significantly higher recruitment of B cells to the thymus of Tg mice as shown on pictures (Figure 4C), by cell counting on thymic sections (Figure 4D) and by flow cytometry (Figure 4E).

**Figure 5:**
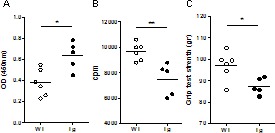
Effects of prolonged Poly(I:C) injections in Tg mice C57BL/6 mice were injected (i.p.) with 200μg of Poly(I:C) or physiological water twice a week for 6 weeks. **A.** Measurement of forelimb grip strength of mice with a grip strength apparatus. **B.** Quantification of 125I-α-bungarotoxin binding to measure AChR density on mouse diaphragm muscle. For each mouse a mean value of labeling per biopsy was calculated. **C.** ELISA for anti-AChR antibodies was performed on the serum after 6 weeks of Poly(I:C) injections. Mann-Whitney test and only *p*-values < 0.05 are indicated (*p < 0.05; **p < 0.01).

As prolonged Poly(I:C) injections are known to induce MG symptoms in WT mice [24], we wondered whether Tg mice were more susceptible to MG and whether thymic B-cell recruitment associated with GC development could be observed. Prolonged Poly(I:C) injections did not further increase B cells in the thymus and by immunohistochemistry, we did not observe the presence of GCs (data not shown). However, Tg mice were significantly more susceptible to develop MG symptoms than WT mice. Indeed, Tg mice displayed higher titers of AChR autoantibodies (Figure 5A) and decreased levels of AChR on muscle diaphragms (5B) than WT mice. Tg mice were also weaker on the grip test apparatus (Figure 5C).

We thus showed that under inflammatory condition, thymic CXCL13 overexpression was capable to trigger an efficient B-cell recruitment to the thymus. Even if we could not observe GC development, we demonstrated that Tg mice were more susceptible to develop MG symptoms upon Poly(I:C) injections.

### Immunization with AChR induced severe EAMG symptoms in Tg mice

An experimental model of MG exists since the seventies [26]. It is induced by immunizing mice 2 to 3 times with an emulsion of T-AChR in CFA (complete Freund's adjuvant). In this model, mice develop antibodies against T-AChR which target AChR on muscle cells. Even though this model is relevant to study muscle weakness caused by the anti-AChR antibody attack, it does not completely recapitulate the human disease, as the thymus is not implicated in this model [27]. Moreover, not all treated animals get sick [28]. We therefore investigated if K5-CXCL13 Tg mice were more susceptible to EAMG and if the thymic overexpression of CXCL13 could trigger thymic changes, such as B-cell hyperplasia as observed in the human disease.

**Figure 6:**
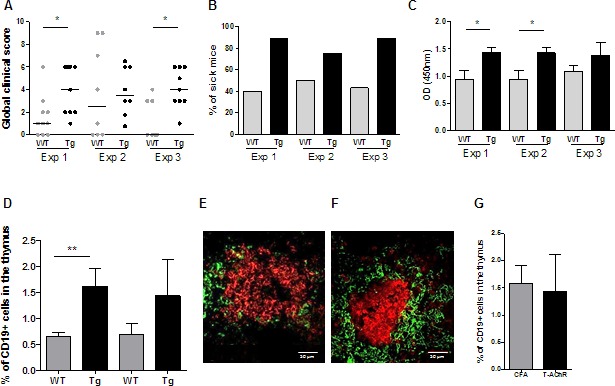
EAMG evaluation for Tg compared to WT mice in three independent experiments Data from 3 independent experiments comparing the susceptibility of C57BL/6 WT (***n*** = 7-10) and K5-CXCL13 Tg (***n*** = 8-9) mice to EAMG. Mice were immunized with T-AChR/CFA twice (experiments 2 and 3) or three times (experiment 1) at 4 week interval. **A.** A global clinical score for each mouse was calculated taking into account the weight loss, the grip test, the inverted grid test, and T-AChR immunized mice were compared to control CFA group mice. Mice considered too sick were euthanized and classified with a global clinical score of 9 in the graph. **B.** The percentages of sick mice (with a global clinical score of at least 2) are shown in kinetic. **C.** ELISAs for anti-AChR antibodies were done on serum taken 2 weeks after the last immunization. **D.** For 2 experiments, thymuses were analyzed by flow cytometry for the percentage of B cells (CD19+ cells) in WT and Tg mice. **E.**-**F.** Representative pictures of B-cell clusters in the thymus of Tg mice with B cells stained with an anti-K5-FITC antibody (green) and a biotinylated anti-B220 antibody plus a streptavidin Alexa-Fluor-594 (red) (E, PBS/CFA control and F, T-AChR/CFA mice). **G.** Flow cytometry analysis of the percentage of thymic B cells (CD19+ cells) in Tg mice for PBS/CFA and T-AChR/CFA immunized mice. p-values were assessed by the Mann-Whitney test and only *p*-values < 0.05 are indicated (*p < 0.05; **p < 0.01).

Three independent experiments were carried out and results are shown in Figure 6 and details for a representative experiment are shown in the supplemental Figure S4. Taking into account the loss of weight, the grip test and the grid test, we calculated a global clinical score (see details in supplemental table S1). Using this global clinical score, we clearly observed that Tg mice were more susceptible to EAMG (Figure 6A and supplemental Figure S4B-S4C) with more than 75% of the Tg mice being sick (with a global clinical score over 2) compared to only 40-50% for the WT mice (Figure 6B). Tg mice were not only sicker, but also had more circulating anti-AChR antibodies in the serum, as assessed by ELISA (Figure 6C).

We analyzed the thymus in 2 experiments for which mice were sacrificed 3 weeks after the second immunization. By flow cytometry, we detected more thymic B cells in Tg mice compared to WT mice (Figure 6D). Moreover, by immunohistochemistry we observed the presence of B-cell clusters in the thymus of a few Tg mice but never in WT mice (Figures 6E-6F). These clusters could correspond to GC-like structures. Their presence was not specific of T-AChR immunizations as we could also observe them in CFA-immunized Tg mice. Moreover, by flow cytometry, we observed a similar increase of B cells in CFA or CFA/T-AChR immunized mice (Figure 6G). In order to determine if the observed B-cell clusters were not residual GCs, we analyzed a few thymuses only 10 days after the boost but similarly we only observed small clusters of B cells (data not shown).

Altogether these data showed that after immunization with T-AChR, Tg mice were clearly more susceptible to MG. The symptoms went along with an elevated level of anti-AChR serum antibodies and the formation of thymic B-cell clusters in a certain number of Tg mice.

## DISCUSSION

The opening question of our work was to determine whether CXCL13 could trigger B-cell recruitment to peripheral organs and lead to ectopic GC development, such as observed in tertiary lymphoid tissues, such as in the thymus of MG patients. Consequently, we created a novel Tg mouse model with a K5 driven expression of CXCL13 in order to induce its overexpression in the thymus and to use this mouse model for studies related to MG.

### Consequences of CXCL13 overexpression in the thymus

Previous studies have clearly demonstrated that CXCL13 plays a central role in early-onset MG. CXCL13 is overexpressed by medullary TECs of MG patients and B-cell attraction to thymic extracts is blocked *in vitro* by anti-CXCL13 antibodies. Moreover, the levels of thymic CXCL13 correlate with disease severity [17, 19, 20]. Our results strongly indicated that thymic overexpression of CXCL13 by itself was not capable of inducing the B-cell related changes associated with MG, as naïve Tg mice did not show increased number of thymic B cells nor the development of GCs.

In parallel, we observed increased levels of CXCL13 in the serum of Tg mice similar to increased CXCL13 serum levels in autoimmune diseases associated with cell infiltrations in divers organs and even ectopic GCs [19, 29]. High CXCL13 serum could hold back CXCR5^+^ cells in the blood and prevent their migration to peripheral organs. However, It is known that cells migrate preferentially to gradient chemokines displayed by tissues (chemotaxis) rather than being randomly attracted to circulating chemokines by chemokinesis [30]. Here, even with high levels of circulating CXCL13 in Tg mice, we observed a decreased percentage of CD19^+^CXCR5^+^ and CD4^+^CXCR5^+^ cells in the blood suggesting their recruitment to peripheral organs.

But why was no recruitment of peripheral B cells observed in the thymus of Tg mice despite the high thymic CXCL13 levels? In the literature, induced overexpression of chemokines in the thymus is described in models for which the transgene expression is driven by a T cell-specific lck promoter [31, 32] while chemokine overexpression induced in thymic stromal cells are hardly described. CCL2 expression under the myelin basic promoter leads to the chemokine overexpression by thymic endothelial cells that can directly drive plasmacytoid dendritic cell recruitment [33]. This result is encouraging as it demonstrates the feasibility of “forcing” the recruitment of peripheral cells to the thymus. The effect of chemokine overexpression may not only depend on the cell type that overexpresses the chemokine but can also depend on targeted tissues and the environment. While e.g. CCL21 triggers tertiary lymphoid organ formation in the pancreas and the thyroid, it fails to do so in the brain and skin [34, 35, 36]. Likewise, CXCL13 overexpression in β cells of the pancreatic islets causes high endothelial venule development and recruitment of dendritic cells, B and T cells [37] while enhanced CXCL13 expression in gut epithelial cells supports mobilization of B cells, NK cells and lymphoid tissue inducer cells with an increase in the number of intestinal lymphoid follicles [38]. The different outcomes of CXCL13 overexpression in pancreas, gut and thymus may be explained by the chemical and cellular microenvironment which influences the effects of chemokine.

### Inflammation catalyzes CXCL13 chemotatic effects

The overexpression of CXCL13 by itself in K5-CXCL13 Tg mice was not sufficient for inducing MG related thymic changes. However, the induction of a systemic inflammation by Poly(I:C) triggered a strong B-cell trafficking to the thymus. The effects of Poly(I:C) seemed to synergize with the already high expression of CXCL13 in Tg mice and drive B cells into the thymus. Pathogen infections are suspected to induce MG in susceptible patients [39]. Inflammation subsequent to pathogen infection appears to be a key event to optimize the recruitment of mature lymphocytes to peripheral organs [40] and even in the thymus [41]. Poly(I:C) a synthetic molecule mimicking dsRNA from viral infections is capable of triggering thymic events related to MG through the intra-thymic overexpression of IFN-β [24]. IFN-I that is released during pathogen infection could favor cell motility [42]. It could also induce the overexpression of different chemokines, such as CCL21 that might also be involved in B-cell recruitment and GC development [25, 23]. In fact, upon infection the induced-expression of CXCL13 together with other chemokines, such as CCL21 and CCL19, could be indispensable to induce ectopic GC development [43]. Pathogen infection or inflammation could also be crucial in other Tg mouse models with specific chemokine expression. Indeed, cerebral overexpression of CCL2 leads to cell infiltrations, which are enhanced by lipopolysaccharide (LPS) treatment supporting the idea of a synergizing action [44]. Similarly, CCL21 expression in the central nervous system (CNS) augments the migration of CD4-T cells from perivascular spaces into the CNS parenchyma following toxoplasma gondii infection of mice [45].

Even though the combination of CXCL13 overexpression and Poly(I:C) injections triggered the migration of B cells to the thymus, it did not lead to the formation of GCs. Luther *et al* on the other hand had shown that CXCL13 overexpression in the pancreas induces its transformation into a lymphoid organ with recruitment of lymphocytes, development of GCs and the formation of high endothelial venules. They demonstrated that these changes were strongly dependent on B cells and lymphotoxin [37]. In addition, Litsiou *et al.* observed that lungs of patients with chronic obstructive pulmonary disease are also characterized by high levels of CXCL13 and ectopic GC development. They proposed a mechanism of action where CXCL13 promotes B-cell migration to ectopic sites, and creates a positive feedback loop by up-regulating lymphotoxin on B cells, which in turn further induces CXCL13 [46]. In our experiments, we did not observe more lymphotoxin alpha and beta mRNA in Tg and WT upon Poly(I:C) injections (data not shown). Altogether these data suggest that ectopic GC development might require the recruitment of larger amount of B cells to initiate the process of transformation into a tertiary lymphoid organ.

### K5-CXCL13 Tg mice, a model mimicking the human MG

The current EAMG model relies on multiple injections of purified T-AChR in CFA which leads to the production of anti-AChR antibodies and muscular symptoms as for MG patients [28]. The main drawback of this model is that it does not show any thymic abnormalities as in the human disease [27]. The Tg mice that we generated have more in common with human MG than the current EAMG model. It displayed CXCL13 overproduction in TECs and it showed an increased serum level of CXCL13 [17, 19, 20]. When we immunized with T-AChR, the number of Tg mice that became sick was higher than for WT mice and they showed more severe clinical signs and higher anti-AChR antibody titers. Under these conditions, after the last immunization we could detect thymic B-cell aggregates in some mice that could correspond to GC-like structures. These lymphoid structures were observed in a few T-AChR immunized mice as well as in CFA immunized Tg mice. This effect was independent of the T-AChR and could only be related to the inflammation induced by CFA injections. We also analyzed the thymic mRNA levels for lymphotoxin alpha and beta but did not observe any increases upon CFA immunization with or without T-AChR suggesting that lymphotoxin might not be indispensable for thymic GC development (Supplemental Figures S4E-S4F), as for the human disease (data not shown).

Here, we present an alternative EAMG model that better mimics the human disease. Even if we did not observe GCs in all CFA-induced mice, in human not all MG patients display GCs eithers [16].

In summary, by using a novel transgenic mouse model with a thymic overexpression of CXCL13, we demonstrated that CXCL13 by itself was not sufficient to induce peripheral B-cell recruitment to the thymus. However, in inflammatory conditions, CXCL13 triggered a strong recruitment of B cells to the thymus. In the experimental mouse MG model, we demonstrated that the thymic CXCL13 overexpression rendered Tg mice more susceptible to experimental MG and could even induce GC-like structures. Consequently, the generation of the Tg K5-CXCL13 mice has allowed us to better define the role of CXCL13 in the pathophysiology of MG and to develop a new EAMG model more relevant to the human MG disease. This mouse model is also of great interest to test novel therapeutic approaches targeting CXCL13-CXCR5 interactions to avoid ectopic GC development [47, 48].

## MATERIALS AND METHODS

### Vector construction and verification

A keratin 5 (K5) promoter driven mouse CXCL13 transgene (K5-CXCL13) was prepared in order to induce CXCL13 expression in medullary TECs. A pEYFP 1 plasmid expressing the enhanced green fluorescent protein (EGFP) gene under the bovine K5 promoter was kindly provided by Prof Daniel Aberdam (INSERM, France). The EGFP gene was then replaced with the full-length cDNA of mouse CXCL13 by the cloning service of GeneCust Europe (Dudelang, Luxembourg) (Supplemental Figure S1A). Functionality of K5-mCXCL13 vector was verified by transfection of the human epithelial cell line HaCat known to express keratin 5. Two days after transfection, cells were analyzed for CXCL13 at the mRNA and protein level. We demonstrated by PCR, ELISA and immunohistochemistry that transfected cells expressed murine CXCL13, which confirmed the functionality of the K5-mCXCL13 plasmid (Supplemental Figure S1B-S1D).

### Transgenesis

For microinjection, the K5-CXCL13 transgene flanked by Not1 restriction site was separated from the vector by digestion with restriction enzymes Not1 (Promega, Charbonnieres, France) followed by gel-separation and purification with QIAquick gel extraction kit (Invitrogen, Villebon sur Yvette, France). The size of the isolated construct was verified by gel-electrophoresis.

The injection of the linearized K5-CXCL13 transgene into fertilized oocytes from C57BL/6 mice was performed according to the standard protocol of the transgenesis facility of the Weizmann institute (Rehovot, Israel). About 200 microinjected zygotes were transferred to 20 pseudo-pregnant females resulting in two founder mice which were backcrossed to C57BL/6 WT mice. The transgene was detected by PCR on DNA extracted from tail tissue using the following primers: 5′-GCTGAAGTCCCTGAAGCAAG (K5, forward) and GTATTCTGGAAGCCCAT (CXCL13, reverse). Homozygote mice in the third and fourth generation with high levels of K5-CXCL13 transgene were identified by quantitative PCR. Homozygosity was confirmed by crossings between Tg and WT mice resulting in offsprings, which were all Tg.

For the following experiments, K5-CXCL13 C57BL/6 female mice were transferred and breed in a SPF animal care facility (CEF - Pierre and Marie Curie University, Paris, France). C57BL/6 female mice were purchased from Janvier Labs (Saint-Berthevin, France). The study was approved by the local Ethics Committee (agreement n° 2569.01).

### Quantitative RT-PCR

Total RNA was extracted as previously described and 1μg of RNA was reverse transcribed for 1h at 42°C using AMV (Roche Applied Science, Mannheim, Germany) with oligo-dT (Invitrogen). RT-PCR reactions were performed with the LightCycler^®^ 480 System [25]. The primer sequences (Eurogentec, Angers, France) are listed in table S2. All samples were normalized to GAPDH.

### CXCL13 ELISA

Antibodies and recombinant CXCL13 were purchased from R&D Systems. CXCL13 (AF470) antibody was diluted at 1 μg/ml in coating buffer and incubated overnight at 4°C. 100μl of thymic extracts (0.5 μg in PBS), serum samples (1/50,000 or 1/200,000 in PBS) or CXCL13 standard (470-BC-025) were incubated for 90 min at 37°C and wells were washed. Subsequently, 0.20 μg/ml of biotinylated anti-CXCL13 (BAF470), and streptavidin-horseradish peroxidase (HRP, Beckman Coulter, Villepinte, France) were used to detect CXCL13. Tetramethylbenzedine was used for color development and plates were read at 450 nm on a MRX-microplate reader (DYNEX Technologies, ThermoLabsystems, Cergy-Pontoise, France).

### Immunohistochemistry

Cryosections of thymic samples (7μm) were fixed in ice-cold acetone for 20 minutes and unspecific binding sites blocked with 2% BSA. Sections were either stained with hematoxylin for light microscopy or with fluorescently labeled antibodies for fluorescent microscopy: anti-K5-FITC antibody (AF138, Eurogentec) for medullary thymic epithelial cells, while B cells were detected with a biotinylated anti-B220 antibody (553085, BD bioscience) and streptavidin Alexa-Fluor-594 (S11227, Invitrogen). Images were acquired with a ZeissAxio Observer Z1 Inverted Microscope. The number of B cells was counted on entire thymic sections and normalized by the size of the sections, which was assessed with the AxioVision software.

### Flow cytometry

Single cell suspensions from thymus and spleen were prepared by passing the organs through a nylon mesh. For spleen and blood samples, erythrocytes were removed using lysis buffer from BD Bioscience (Le Pont de Claix, France). Isolated cells were incubated for 5 minutes on ice with Fc-block (BD Bioscience) to reduce unspecific binding of antibodies to Fc-receptor. Cells were incubated for 30 minutes on ice with antibodies from BD Bioscience, except when specified: anti-CD19 PE (553786) or CD19-efluor450 (48-0193-82, eBioscience, Paris, France), anti-CD4 Alexa700 (557956) or anti-CD4-APC (553051), anti-CD8a PE-Cy7 (552877), anti-CXCR5 (12-7185-80, eBioscience). Flow cytometry was performed on a FACS Verse (BD Biosciences).

### Poly(I:C) injections in mice

As described in Cufi *et al.* [24], 6 week-old C57BL/6 mice were injected (i.p.) with 200μg of Poly(I:C) or physiological water. For short-term experiments, mice were injected three times every other day and sacrificed at day 6. For long-term experiments, mice were injected twice a week. Mice were monitored with the grip test apparatus (Bioseb-Bio-GS3) and sacrificed after 6 weeks. Serum anti-AChR antibodies were measured by ELISA as described below. AChR density on mouse diaphragms was quantified as described before [24]. Briefly, diaphragms were stained for acetylcholinesterase with a classical Koelle reaction. Eight calibrated biopsies were taken out along the end-plate location. Labelling was done for 2 hours with ^125^I-α-bungarotoxin in HBSS-BSA 0.5%. Biopsies were washed extensively and radioactivity was determined on a gamma counter.

### Experimental autoimmune myasthenia gravis (EAMG) model

6-8 week-old C57BL/6 mice were immunized with purified *Torpedo californica* AChR (T-AChR) prepared as described by Aharonov et al. [49]. T-AChR was emulsified with an equal volume of CFA (Sigma, Saint Quentin Fallavier, France) supplemented with mycobacterium tuberculosis 10 mg/ml (H37RA, BD Difco, Villepinte, France). Mice were subcutaneously injected (200 μl/mouse, 30 μg AChR) at several sites (hind foot-pads, tail base and in the back). After 4 weeks, mice were immunized a second time with a T-AChR and CFA emulsion. A third immunization was done if the percentage of sick mice was insufficient. Mice were euthanized 3 weeks after the last immunization for assessment of immunopathological parameters. Control mice were injected similarly with CFA emulsion devoid of T-AChR [28].

Mice were regularly monitored for signs of muscle weakness and mice that were too weak were euthanized. Different tests for clinical evaluation were done 1 week before the second immunization and 2 weeks after.

### Clinical evaluation for EAMG experiments

Different assessments were taken into account to evaluate the clinical state of the animals. Mice were weighed. Muscle strength was analyzed by measuring the forelimb strength with a grip strength apparatus. As clinical signs are not always obvious in resting mice, the grip test measurements were done after a 3-minute run on a treadmill. Because Tg mice were slightly weaker compared to WT mice, a mean value was calculated for the CFA control group for each mouse strain, and a score was attributed to each mouse after normalization to the CFA control group. An inverted grid test was also carried out. Mice were tired by gently dragging them 20 times across the top grid of a cage and then carefully observed as the grid was rotated. A global clinical score was then calculated (see details in supplemental table S1) taking into account the loss of weight, the grip test and the grid test for T-AChR immunized mice compared to control mice. A mouse was considered sick when it reached a global clinical score of 2.

### Anti-AChR antibody ELISA

96-well ELISA plates were coated overnight at 4°C with 1 μg/ml of T-AChR diluted in 10 mM NaHCO3 buffer, pH 9.6. T-AChR coated wells were blocked with 10% SVF in PBS at 37°C for 2-3 hours. 100 μl of mouse serum (1/100,000) per well were incubated for 90 min at 37°C. Subsequently, wells were washed 4 times with the PBS-Tween buffer. 100 μl of 1/10,000 diluted biotinylated anti-mouse IgGs (E0413, Dako, Courtaboeuf, France) were added for 90 min at 37°C. Next, samples were incubated with 100 μl of streptavidin-horseradish peroxidase 1/20,000 (PN IM0309, Beckman Coulter, Villepinte, France) for 30 minutes and tetramethylbenzedine was used for color development. To determine the optical density (OD) at 450 nm, we used a microtiter plate reader spectrophotometer.

### Statistical analyses

In bar graphs, results are expressed as mean of different experiments. Error bars represent SEM. For 2-by-2 comparisons, non-parametric Mann-Whitney test was applied as specified in figure legends.

## SUPPLEMENTARY MATERIAL TABLES AND FIGURES



## Abbreviations

AChR: acetylcholine receptor
CFA: complete Freund's adjuvant
EAMG: experimental autoimmune myasthenia gravis
GC: germinal center
K5: keratin 5
MG: myasthenia gravis
Poly(I:C): polyinosinic-polycytidylic acid
TEC: thymic epithelial cell
Tg: transgenic
TFH: follicular helper T
WT: wildtype

## References

[1] Gunn MD, Tangemann K, Tam C, Cyster JG, Rosen SD, Williams LT (1998). A chemokine expressed in lymphoid high endothelial venules promotes the adhesion and chemotaxis of naive T lymphocytes. Proc Natl Acad Sci U S A.

[2] Legler DF, Loetscher M, Roos RS, Clark-Lewis I, Baggiolini M, Moser B (1998). B cell-attracting chemokine 1, a human CXC chemokine expressed in lymphoid tissues, selectively attracts B lymphocytes *via* BLR1/CXCR5. J Exp Med.

[3] Forster R, Mattis AE, Kremmer E, Wolf E, Brem G, Lipp M (1996). A putative chemokine receptor, BLR1, directs B cell migration to defined lymphoid organs and specific anatomic compartments of the spleen. Cell.

[4] Ansel KM, Ngo VN, Hyman PL, Luther SA, Forster R, Sedgwick JD, Browning JL, Lipp M, Cyster JG (2000). A chemokine-driven positive feedback loop organizes lymphoid follicles. Nature.

[5] Shi K, Hayashida K, Kaneko M, Hashimoto J, Tomita T, Lipsky PE, Yoshikawa H, Ochi T (2001). Lymphoid chemokine B cell-attracting chemokine-1 (CXCL13) is expressed in germinal center of ectopic lymphoid follicles within the synovium of chronic arthritis patients. J Immunol.

[6] Weiss JM, Cufi P, Le Panse R, Berrih-Aknin S (2013). The thymus in autoimmune Myasthenia Gravis: Paradigm for a tertiary lymphoid organ. Rev Neurol (Paris).

[7] Allen CD, Ansel KM, Low C, Lesley R, Tamamura H, Fujii N, Cyster JG (2004). Germinal center dark and light zone organization is mediated by CXCR4 and CXCR5. Nat Immunol.

[8] Hardtke S, Ohl L, Forster R (2005). Balanced expression of CXCR5 and CCR7 on follicular T helper cells determines their transient positioning to lymph node follicles and is essential for efficient B-cell help. Blood.

[9] Barone F, Bombardieri M, Manzo A, Blades MC, Morgan PR, Challacombe SJ, Valesini G, Pitzalis C (2005). Association of CXCL13 and CCL21 expression with the progressive organization of lymphoid-like structures in Sjogren's syndrome. Arthritis Rheum.

[10] Khademi M, Kockum I, Andersson ML, Iacobaeus E, Brundin L, Sellebjerg F, Hillert J, Piehl F, Olsson T (2011). Cerebrospinal fluid CXCL13 in multiple sclerosis: a suggestive prognostic marker for the disease course. Mult Scler.

[11] Aharonov A, Abramsky O, Tarrab-Hazdai R, Fuchs S (1975). Humoral antibodies to acetylcholine receptor in patients with myasthenia gravis. Lancet.

[12] Le Panse R, Bismuth J, Cizeron-Clairac G, Weiss JM, Cufi P, Dartevelle P, De Rosbo NK, Berrih-Aknin S (2010). Thymic remodeling associated with hyperplasia in myasthenia gravis. Autoimmunity.

[13] Mesnard-Rouiller L, Bismuth J, Wakkach A, Poea-Guyon S, Berrih-Aknin S (2004). Thymic myoid cells express high levels of muscle genes. J Neuroimmunol.

[14] Safar D, Berrih-Aknin S, Morel E (1987). *In vitro* anti-acetylcholine receptor antibody synthesis by myasthenia gravis patient lymphocytes: correlations with thymic histology and thymic epithelial-cell interactions. J Clin Immunol.

[15] Melms A, Schalke BC, Kirchner T, Muller-Hermelink HK, Albert E, Wekerle H (1988). Thymus in myasthenia gravis. Isolation of T-lymphocyte lines specific for the nicotinic acetylcholine receptor from thymuses of myasthenic patients. J Clin Invest.

[16] Berrih-Aknin S, Morel E, Raimond F, Safar D, Gaud C, Binet J, Levasseur P, Bach J (1987). The role of the thymus in myasthenia gravis: immunohistological and immunological studies in 115 cases. Ann N Y Acad Sci.

[17] Méraouna A, Cizeron-Clairac G, Le Panse R, Bismuth J, Truffault F, Talaksen C, Berrih-Aknin S (2006). The chemokine CXCL13 is a key molecule in autoimmune Myasthenia Gravis. Blood.

[18] Le Panse R, Cizeron-Clairac G, Bismuth J, Berrih-Aknin S (2006). Microarrays reveal distinct gene signatures in the thymus of seropositive and seronegative myasthenia gravis patients and the role of CC chemokine ligand 21 in thymic hyperplasia. J Immunol.

[19] Shiao YM, Lee CC, Hsu YH, Huang SF, Lin CY, Li LH, Fann CS, Tsai CY, Tsai SF, Chiu HC (2010). Ectopic and high CXCL13 chemokine expression in myasthenia gravis with thymic lymphoid hyperplasia. J Neuroimmunol.

[20] Zhang M, Guo J, Li H, Zhou Y, Tian F, Gong L, Wang X, Li Z, Zhang W (2013). Expression of immune molecules CD25 and CXCL13 correlated with clinical severity of myasthenia gravis. J Mol Neurosci.

[21] Weiss JM, Cufi P, Bismuth J, Eymard B, Fadel E, Berrih-Aknin S, Le Panse R (2013). SDF-1/CXCL12 recruits B cells and antigen-presenting cells to the thymus of autoimmune myasthenia gravis patients. Immunobiology.

[22] Feferman T, Maiti PK, Berrih-Aknin S, Bismuth J, Bidault J, Fuchs S, Souroujon MC (2005). Overexpression of IFN-induced protein 10 and its receptor CXCR3 in myasthenia gravis. J Immunol.

[23] Berrih-Aknin S, Ruhlmann N, Bismuth J, Cizeron-Clairac G, Zelman E, Shachar I, Dartevelle P, de Rosbo NK, Le Panse R (2009). CCL21 overexpressed on lymphatic vessels drives thymic hyperplasia in myasthenia. Ann Neurol.

[24] Cufi P, Dragin N, Weiss JM, Martinez-Martinez P, De Baets MH, Roussin R, Fadel E, Berrih-Aknin S, Le Panse R (2013). Implication of double-stranded RNA signaling in the etiology of autoimmune myasthenia gravis. Ann Neurol.

[25] Cufi P, Dragin N, Ruhlmann N, Weiss JM, Fadel E, Serraf A, Berrih-Aknin S, Le Panse R (2014). Central role of interferon-beta in thymic events leading to myasthenia gravis. J Autoimmun.

[26] Fuchs S, Nevo D, Tarrab-Hazdai R, Yaar I (1976). Strain differences in the autoimmune response of mice to acetylcholine receptors. Nature.

[27] Meinl E, Klinkert WE, Wekerle H (1991). The thymus in myasthenia gravis. Changes typical for the human disease are absent in experimental autoimmune myasthenia gravis of the Lewis rat. Am J Pathol.

[28] Tuzun E, Berrih-Aknin S, Brenner T, Kusner LL, Le Panse R, Yang H, Tzartos S, Christadoss P (2015). Guidelines for standard preclinical experiments in the mouse model of myasthenia gravis induced by acetylcholine receptor immunization. Exp Neurol.

[29] Kramer JM, Klimatcheva E, Rothstein TL (2013). CXCL13 is elevated in Sjogren's syndrome in mice and humans and is implicated in disease pathogenesis. J Leukoc Biol.

[30] Petrie RJ, Doyle AD, Yamada KM (2009). Random *versus* directionally persistent cell migration. Nature reviews Molecular cell biology.

[31] Lira SA, Zalamea P, Heinrich JN, Fuentes ME, Carrasco D, Lewin AC, Barton DS, Durham S, Bravo R (1994). Expression of the chemokine N51/KC in the thymus and epidermis of transgenic mice results in marked infiltration of a single class of inflammatory cells. J Exp Med.

[32] Christopherson KW, Campbell JJ, Hromas RA (2001). Transgenic overexpression of the CC chemokine CCL21 disrupts T-cell migration. Blood.

[33] Cedile O, Lobner M, Toft-Hansen H, Frank I, Wlodarczyk A, Irla M, Owens T (2014). Thymic CCL2 influences induction of T-cell tolerance. J Autoimmun.

[34] Martin AP, Coronel EC, Sano G, Chen SC, Vassileva G, Canasto-Chibuque C, Sedgwick JD, Frenette PS, Lipp M, Furtado GC, Lira SA (2004). A novel model for lymphocytic infiltration of the thyroid gland generated by transgenic expression of the CC chemokine CCL21. J Immunol.

[35] Chen SC, Vassileva G, Kinsley D, Holzmann S, Manfra D, Wiekowski MT, Romani N, Lira SA (2002). Ectopic expression of the murine chemokines CCL21a and CCL21b induces the formation of lymph node-like structures in pancreas, but not skin, of transgenic mice. J Immunol.

[36] Chen SC, Leach MW, Chen Y, Cai XY, Sullivan L, Wiekowski M, Dovey-Hartman BJ, Zlotnik A, Lira SA (2002). Central nervous system inflammation and neurological disease in transgenic mice expressing the CC chemokine CCL21 in oligodendrocytes. J Immunol.

[37] Luther SA, Lopez T, Bai W, Hanahan D, Cyster JG (2000). BLC expression in pancreatic islets causes B cell recruitment and lymphotoxin-dependent lymphoid neogenesis. Immunity.

[38] Marchesi F, Martin AP, Thirunarayanan N, Devany E, Mayer L, Grisotto MG, Furtado GC, Lira SA (2009). CXCL13 expression in the gut promotes accumulation of IL-22-producing lymphoid tissue-inducer cells, and formation of isolated lymphoid follicles. Mucosal immunology.

[39] Cavalcante P, Serafini B, Rosicarelli B, Maggi L, Barberis M, Antozzi C, Berrih-Aknin S, Bernasconi P, Aloisi F, Mantegazza R (2010). Epstein-Barr virus persistence and reactivation in myasthenia gravis thymus. Ann Neurol.

[40] Katzman SD, Fowell DJ (2008). Pathogen-imposed skewing of mouse chemokine and cytokine expression at the infected tissue site. J Clin Invest.

[41] Hodge DL, Reynolds D, Cerban FM, Correa SG, Baez NS, Young HA, Rodriguez-Galan MC (2012). MCP-1/CCR2 interactions direct migration of peripheral B and T lymphocytes to the thymus during acute infectious/inflammatory processes. Eur J Immunol.

[42] Foster GR, Masri SH, David R, Jones M, Datta A, Lombardi G, Runkell L, de Dios C, Sizing I, James MJ, Marelli-Berg FM (2004). IFN-alpha subtypes differentially affect human T cell motility. J Immunol.

[43] Rangel-Moreno J, Moyron-Quiroz JE, Hartson L, Kusser K, Randall TD (2007). Pulmonary expression of CXC chemokine ligand 13, CC chemokine ligand 19, and CC chemokine ligand 21 is essential for local immunity to influenza. Proc Natl Acad Sci U S A.

[44] Rodriguez-Gallego E, Riera-Borrull M, Hernandez-Aguilera A, Marine-Casado R, Rull A, Beltran-Debon R, Luciano-Mateo F, Menendez JA, Vazquez-Martin A, Sirvent JJ, Martin-Paredero V, Corbi AL, Sierra-Filardi E, Aragones G, Garcia-Heredia A, Camps J (2013). Ubiquitous transgenic overexpression of C-C chemokine ligand 2: a model to assess the combined effect of high energy intake and continuous low-grade inflammation. Mediators Inflamm.

[45] Ploix CC, Noor S, Crane J, Masek K, Carter W, Lo DD, Wilson EH, Carson MJ (2011). CNS-derived CCL21 is both sufficient to drive homeostatic CD4+ T cell proliferation and necessary for efficient CD4+ T cell migration into the CNS parenchyma following Toxoplasma gondii infection. Brain Behav Immun.

[46] Litsiou E, Semitekolou M, Galani IE, Morianos I, Tsoutsa A, Kara P, Rontogianni D, Bellenis I, Konstantinou M, Potaris K, Andreakos E, Sideras P, Zakynthinos S, Tsoumakidou M (2013). CXCL13 production in B cells *via* Toll-like receptor/lymphotoxin receptor signaling is involved in lymphoid neogenesis in chronic obstructive pulmonary disease. Am J Respir Crit Care Med.

[47] Finch DK, Ettinger R, Karnell JL, Herbst R, Sleeman MA (2013). Effects of CXCL13 inhibition on lymphoid follicles in models of autoimmune disease. Eur J Clin Invest.

[48] Klimatcheva E, Pandina T, Reilly C, Torno S, Bussler H, Scrivens M, Jonason A, Mallow C, Doherty M, Paris M, Smith ES, Zauderer M (2015). CXCL13 antibody for the treatment of autoimmune disorders. BMC Immunol.

[49] Aharonov A, Tarrab-Hazdai R, Silman I, Fuchs S (1977). Immunochemical studies on acetylcholine receptor from Torpedo californica. Immunochemistry.

