# Supplementary material for: Novel CXCL13 transgenic mouse: inflammation drives pathogenic effect of CXCL13 in experimental myasthenia gravis
Source: Oncotarget. 2016 Jan 11;7(7):7550–62. doi: 10.18632/oncotarget.6885 (PMC4884937; doi:10.18632/oncotarget.6885)
Supplement: Supplementary file 1 [file oncotarget-07-7550-s001.pdf]

Novel CXCL13 transgenic mouse: inflammation drives pathogenic effect of CXCL13 in experimental myasthenia gravis

Supplementary Material

Table S1: Global clinical score evaluation

The global clinical score was graded on 9 taking into account 3 measures: the weight loss, the muscle strength and an inverted grid test. Each of these measures was graded on 3 as detailed in the table below. The grip test measurements were done after a 3 minutes run on a treadmill. For the weight and the grip test analyses, a mean value was calculated for the CFA control group for each mouse strain, and a score was attributed to each mouse compared to the mean value. For the inverted grid test, mice were first tired by gently dragging them across a grid 20 times. Immediately after, the grid was rotated to the inverted position and held steadily for more than 60 seconds. During this time lapse, mice were carefully observed to detect any sign of abnormal behavior. A mice reaching a global clinical score of 9 was considered too sick and euthanized.

Score	Weight	Grip test (T-AChR vs. CFA group)	Inverted grid test
0	Less than 5% weight loss	Less than 5% decrease	No muscle weakness<br>Hang more than 60 sec
1	Between 5-10% weight loss	Between 5-15% decrease	No obvious muscle weakness but less mobile<br>Hang for 45-60 sec
2	Between 10-15% weight loss	Between 15-25 % decrease	Muscle weakness and less<br>Hang for 30-40 sec
3	More than 15 % loss weight	More than 25 % decrease	Reduced mobility<br>Fall before 30 sec

Table S2: List of primers

Primers for mouse genotyping

Gene	Mouse primers	
**CXCL13**	GCTGAAGTCCCTGAAGCAAG	(f)
	GTATTCTGGAAGCCCAT	(r)

Primers for PCR

Genes	Mouse primers	
**CXCL13**	TGAGGCTCAGCACAGCAA	(f)
	ATGGGCTTCCAGAATACCG	(r)
**CCL21**	CCCTGGACCCAAGGCAGT	(f)
	AGGCTTAGAGTGCTTCCGGG	(r)
**CCL19**	CTGCCTCAGATTATCTGCCAT	(f)
	CTTCCGCATCATTAGCACCC	(r)
**CXCL10**	GGATGGCTGTCCTAGCTCTG	(f)
	ATAACCCCTTGGGAAGATGG	(r)
**CXCL12**	GCTCTGCATCAGTGACGGTA	(f)
	TAATTTCGGGTCAATGCACA	(r)
**CD19**	GGGACCTGGACTGTGACCTA	(f)
	AGGACAGCCAAAGTGTGGAG	(r)
**GAPDH**	AACTTTGGCATTGTGGAAGG	(f)
	ACACATTGGGGGTAGGAACA	(r)
**Lymphotoxin alpha**	CACGAGGTCCAGCTCTTTTC	(f)
	AGTGCAAAGGCTCCAAAGAA	(r)
**Lymphotoxin beta**	GGAGCACAGGCTCAGAAAAG	(f)
	CCCCTGGATCTGGTGTAGAA	(r)

Supplemental figure S1

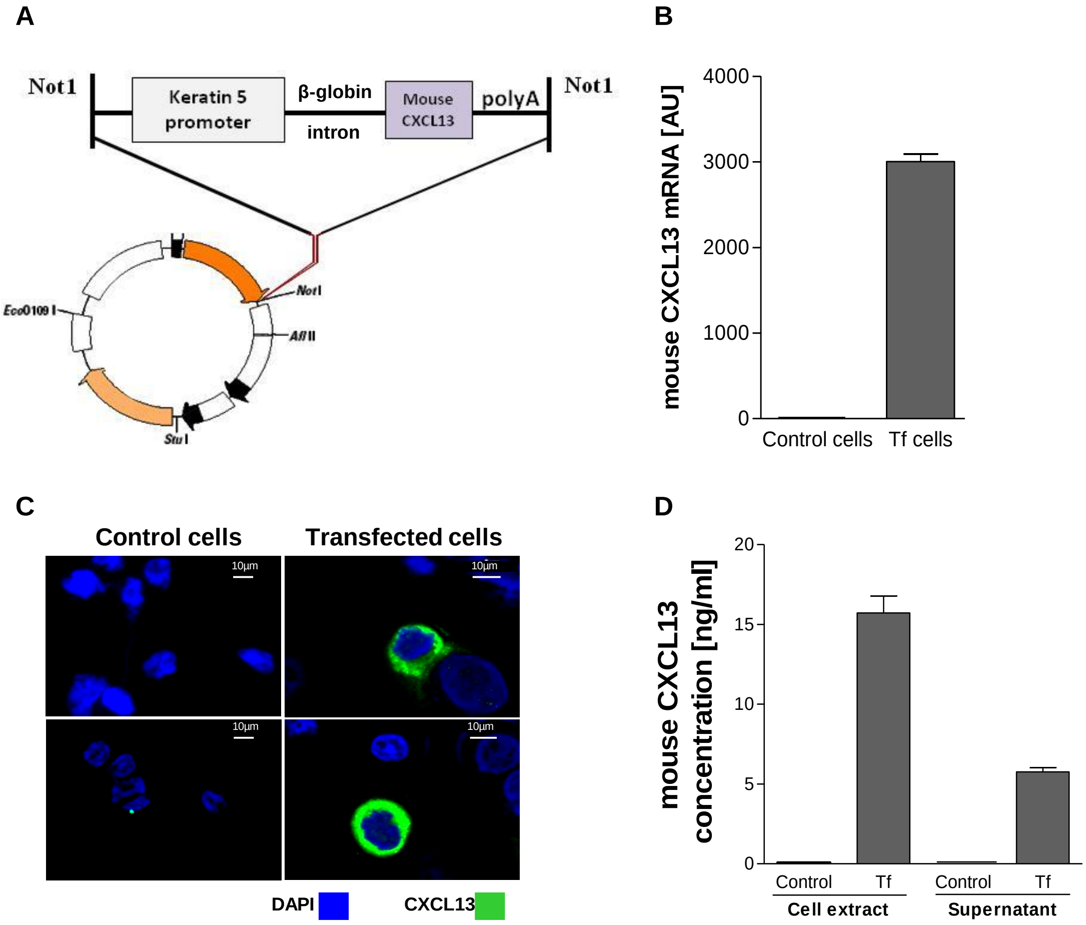

Figure S1: K5-CXCL13 vector led to keratin 5 driven CXCL13 expression.

Scheme of the EYFP-1 plasmid which carries the bovine keratin 5 promoter sequence, a β-globin intron and a poly-A termination sequence (A). Functionality of the K5-CXCL13 vector was verified by transfection of the HaCat human cell line that expresses keratin 5. The plasmid (500 ng) was introduced into the cells by lipofection according to the jetPEI protocol (Polyplus-transfection SA, Illkirch, France). For negative controls, HaCat cells were treated with the transfection reagents alone. Two days later, control and transfected (Tf) cells were harvested and CXCL13 expression was analyzed at the mRNA level by PCR (B) and the protein level by immunohistochemistry (C) and by ELISA (D). The latter was performed on both cell extract and cell supernatant.

Supplemental figure S2

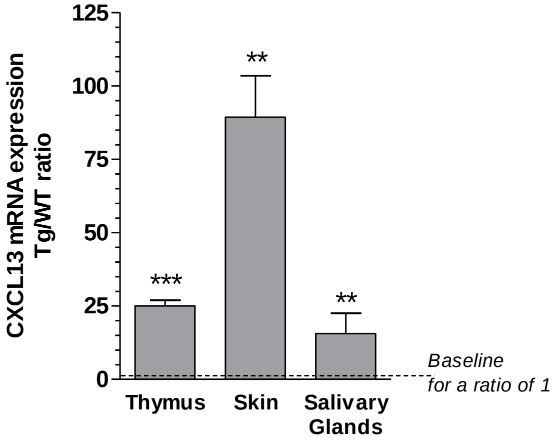

Figure S2: CXCL13 mRNA expression in peripheral organs

We analyzed the expression of CXCL13 mRNA in different organs of transgenic mice compared to WT mice. We selected organs known to express keratin 5 (thymus, skin and salivary glands). Ratios correspond to the CXCL13 mRNA levels in Tg mice (n=21 for thymuses and n=5 for other organs) over the mean level of CXCL13 mRNA in WT mice (mean for 17 thymic samples and 6 for other organs). *p*-values were assessed by the Mann-Whitney test to compare CXCL13 expression in Tg versus WT samples (*p<0.05; **p<0.01; ***p<0.001).

Supplemental figure S3

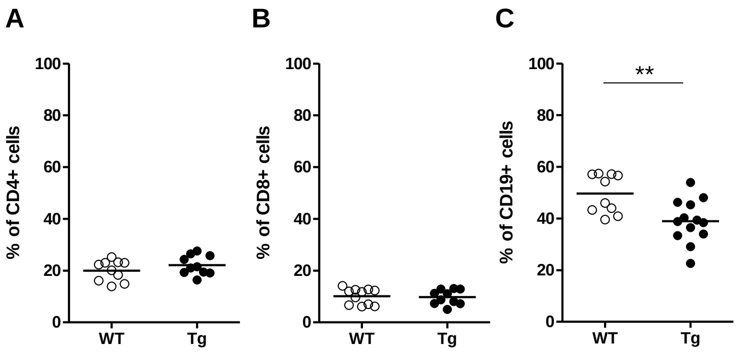

Figure S3: Proportion of circulating lymphoid cells subpopulations in K5-CXCL13 Tg mice.
Blood cells were labeled and analyzed by flow cytometry for CD4 (A), CD8 (B) and CD19 (C). The percentage of cells were analyzed in the lymphocyte gate (determined according to the FSC/SSC characteristic profile of lymphocytes). These analyses were made on 2- to 3-month-old mice. p-values were assessed by the Mann-Whitney test to compare CXCL13 expression in Tg versus WT samples (*p<0.05; **p<0.01; *p<0.05)

Supplemental figure S4

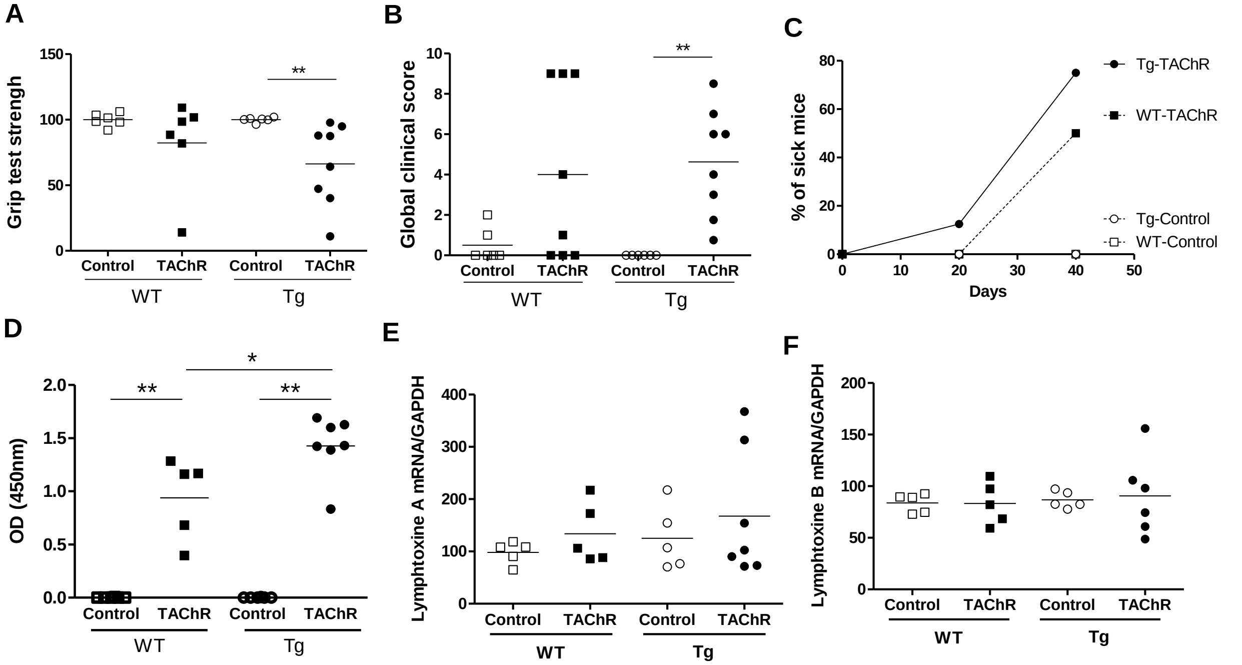

Figure S4. EAMG evaluation for Tg compared to WT mice.

Representative experiment where C57BL6 WT and K5-CXCL13 Tg mice were immunized at day 0 and day 28 with PBS/CFA (controls, n=6) or T-AChR/CFA (EAMG, n=8). Clinical evaluations were done at day 20, 40 and 47 and data obtained at day 47 are shown on graphs A and B. (A) Muscle strength was measured with the grip test apparatus after exercise on the treadmill. For WT and Tg mice, data were normalized separately to the mean (set up at 100 gr) for each CFA control group. (B) A global clinical score for each mouse was calculated taking into account the weight loss, the grip test, the inverted grid test and T-AChR immunized mice were compared to CFA control groups (as detailed in the method section). When a mouse was considered too sick, it was euthanized during the follow-up of the experiment. It was then classified with a global clinical score of 9 in the graph. (C) The percentages of sick mice (with a global clinical score of at least 2) were shown in kinetic along the experiment. (D) ELISA for anti-AChR antibodies. (E-F) Lymphotoxin A and B mRNA expression in the thymus. Data were normalized to GAPDH. p-values were assessed by the Mann-Whitney test and only p-values < 0.05 are indicated (*p<0.05; **p<0.01; ***p<0.001).
